# Regulation of size and scale in vertebrate spinal cord development

**DOI:** 10.1002/wdev.383

**Published:** 2020-05-11

**Authors:** Katarzyna Kuzmicz‐Kowalska, Anna Kicheva

**Affiliations:** ^1^ IST Austria Klosterneuburg Austria

**Keywords:** morphogen, neural tube, pattern scaling, spinal cord, tissue size

## Abstract

All vertebrates have a spinal cord with dimensions and shape specific to their species. Yet how species‐specific organ size and shape are achieved is a fundamental unresolved question in biology. The formation and sculpting of organs begins during embryonic development. As it develops, the spinal cord extends in anterior–posterior direction in synchrony with the overall growth of the body. The dorsoventral (DV) and apicobasal lengths of the spinal cord neuroepithelium also change, while at the same time a characteristic pattern of neural progenitor subtypes along the DV axis is established and elaborated. At the basis of these changes in tissue size and shape are biophysical determinants, such as the change in cell number, cell size and shape, and anisotropic tissue growth. These processes are controlled by global tissue‐scale regulators, such as morphogen signaling gradients as well as mechanical forces. Current challenges in the field are to uncover how these tissue‐scale regulatory mechanisms are translated to the cellular and molecular level, and how regulation of distinct cellular processes gives rise to an overall defined size. Addressing these questions will help not only to achieve a better understanding of how size is controlled, but also of how tissue size is coordinated with the specification of pattern.

This article is categorized under:

Establishment of Spatial and Temporal Patterns > Regulation of Size, Proportion, and Timing
Signaling Pathways > Global Signaling Mechanisms
Nervous System Development > Vertebrates: General Principles

## Graphical abstract

The vertebrate spinal cord changes its shape and size during development. These changes depend on cell behaviours that are regulated by global tissue‐scale mechanisms.

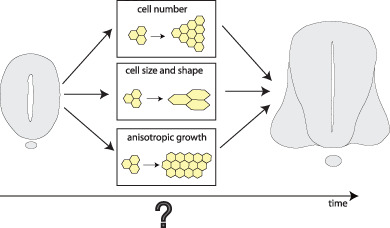

## 1 INTRODUCTION

From the smallest frog, spanning just a few millimeters, to the largest whale reaching more than 30 meters, all vertebrates have a spinal cord that relays somatosensory input between the body and the brain enabling the control of locomotion. Much of these differences in body and organ size in adulthood arise as a result of postembryonic growth. Nevertheless, vertebrate embryos have characteristic dimensions already during early stages of spinal cord formation. How organ size is determined during development is one of the most fundamental unresolved questions in biology. In this review, we discuss how the size of the spinal cord is established and regulated over the course of vertebrate development. We outline distinct aspects of this process and examine how they are interrelated.

**FIGURE 1:**
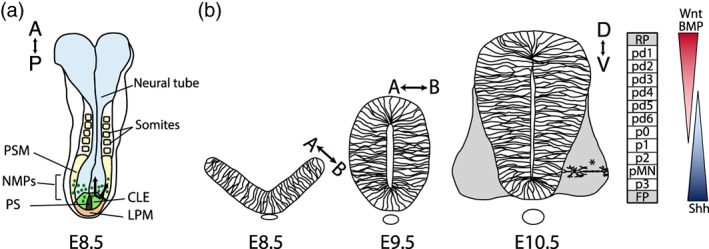
Overview of neural tube development and organization. (a) Top view of a mouse embryo at embryonic day E8.5. Neuromesodermal progenitors (NMPs) in caudal lateral epiblast (CLE) give rise to both neural progenitors and presomitic mesoderm (PSM). Anterior (A) top, posterior (P) bottom. Primitive streak (PS), lateral plate mesoderm (LPM). (b) Transverse sections through the mouse neural tube and underlying notochord at different stages of development. The neural progenitors form a single cell layered pseudostratified epithelium, with their apical side (A) facing the lumen of the neural tube. The dorsal (D) to ventral (V), and apical (A) to basal (B) lengths of the neuroepithelium increase over time. The dorsoventral pattern of progenitor domains pd1 to p3, floor plate (FP), and roof plate (RP) is specified in response to the morphogen gradients of Shh, Wnts and BMPs. Progenitors differentiate into postmitotic neurons (*) that delaminate basally from the epithelium and form a lateral mantle zone (gray)

The spinal cord develops from the posterior part of the neural tube. This tissue originates from neuromesodermal progenitor cells (NMPs) residing in the caudal lateral epiblast (CLE) of the embryo. As the body axis progressively extends towards the posterior, the descendants of NMPs are incorporated into the neural plate and the adjacent paraxial mesoderm (Figure 1a; reviewed in Gouti, Metzis, & Briscoe, 2015; Henrique, Abranches, Verrier, & Storey, 2015. Across all vertebrates, including the lamprey—the most ancient living vertebrate species, the spinal cord consists of an inner epithelial neural progenitor layer (Leung & Shimeld, 2019). This single‐cell layered pseudostratified epithelium is highly proliferative, with the progenitor nuclei undergoing interkinetic movements along the apicobasal (AB) axis in synchrony with the cell cycle (Sauer, 1935). Depending on the species and axial position, the formation of the neural tube occurs either through primary neurulation, where a neural plate folds at the ventral midline, or secondary neurulation, where mesenchymal cells form a solid cord that subsequently cavitates (Gilbert & Barresi, 2016).

As soon as cells adopt neural identity in the neural plate, they are exposed to secreted signals emanating from opposite poles of the tissue: Shh, secreted by the notochord and later also the floor plate, and ligands from the BMP and Wnt families produced by the surface ectoderm and later also the roof plate. Morphogen signaling induces the patterned expression of multiple transcription factors. The combinatorial expression of these transcription factors defines domains of molecularly distinct progenitor subtypes organized along the dorsoventral (DV) axis (Figure 1b) (Briscoe & Small, 2015; Lai, Seal, & Johnson, 2016). Neural progenitors give rise to specific subtypes of neurons and glial cells, which delaminate basally out of the epithelial layer upon their formation (Alaynick, Jessell, & Pfaff, 2011; Rowitch, 2004; Wilcock, Swedlow, & Storey, 2007). Besides their DV identity, neural progenitors possess a regional anterioposterior (AP) identity specified by a Hox code, which is established downstream of Fgf and retinoic acid (RA) gradients along the AP axis (Dasen, 2009).

**FIGURE 2:**
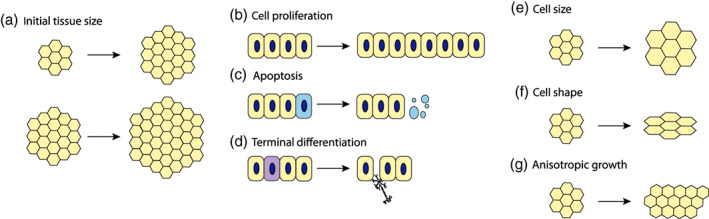
Determinants of tissue size. (a) The final tissue size is determined by the initial size, given similar growth rate. (b) Cell proliferation allows for exponential increase in cell number. (c) Cells can be eliminated through apoptosis leading to a reduction in tissue size. Apoptotic cells become fragmented and are extruded from the epithelium. (d) Progenitors differentiate into postmitotic neurons at a specific rate. Postmitotic neurons delaminate out of the neuroepithelium leading to a reduction in the size of the progenitor domain. (e, f) With time, cells undergo changes in their size (e) and shape (f) altering the absolute dimensions of the epithelium. (g) Tissues can grow at different rates in different directions, leading to changes in tissue shape

Thus, the spinal neuroepithelium has an elaborate underlying molecular pattern and a highly dynamic shape and size during development—it changes along its AP, DV, and AB dimensions, becoming increasingly anisotropic as the body axis extends (Figure 1b). Despite this apparent complexity, the shape and size of the spinal cord during development can be understood in terms of basic mathematical relationships that describe the change in number, the dimensions and the arrangement of cells within the tissue over time. These underlying cell behaviors, regardless of the specific mechanisms by which they are regulated, can all potentially alter tissue size. The number of cells within the tissue is initially determined by the number of progenitors that are incorporated into or specified as the organ primordium. The subsequent expansion of the tissue per unit time depends on the number of cells that are produced by cell proliferation, and lost due to terminal differentiation or cell death. The cell sizes and shapes determine how the number of cells relates to the absolute tissue dimensions. Finally, directional processes controlling cell shapes and the rearrangement of cells in different directions determine the anisotropy of tissue growth and the resulting tissue shape (Figure 2).

These basic determinants of tissue size (Figure 2) are subject to local cellular and molecular regulation, as well as global biochemical and mechanical tissue‐scale control. Understanding how these levels of regulation are linked to produce the emergent species‐specific tissue size and shape is a forthcoming challenge. In this review, we outline the basic determinants of size and shape in the spinal cord and discuss how they are regulated. We focus primarily on regulation by biochemical factors, in particular on morphogen signaling pathways and their downstream targets. We discuss the earliest stages of spinal cord formation, rather than later developmental and postembryonic stages, focusing on amniotes.

## 2 INITIAL POOL OF SPINAL CORD PROGENITORS

Organ development can be strongly dependent on the initial number of cells available for its formation. Different species have evolved distinct mechanisms for coping with alterations in embryo size prior to organogenesis. Similar to amphibians (De Robertis, 2006), zebrafish can sustain removal of a large number of cells at early blastula stages. This results in the formation of size‐reduced individuals with proportionally scaled organs, including a smaller neural tube with scaled pattern (Collins, Ishimatsu, Tsai, & Megason, 2018). By contrast, in mammals the size of the embryo is tightly regulated around the time of gastrulation (Snow, Tam, & McLaren, 1981). Aggregates of two mouse embryos reduce their proliferation rate between E5 and E6, and end up with cell numbers similar to wildtype upon gastrulation (Lewis & Rossant, 1982; Rands, 1986a). At the same stage, wildtype mouse embryos eliminate up to 35% of their epiblast cells, which have lower fitness or insufficiently advanced differentiation, raising the possibility that cell competition plays a role in size regulation at this stage (Bowling et al., 2018; Clavería, Giovinazzo, Sierra, & Torres, 2013; Hashimoto & Sasaki, 2019; Morris, Guo, & Zernicka‐Goetz, 2012; Sancho et al., 2013). Conversely, an experimental reduction in the number of cells leads to increased cell proliferation and embryo growth up to about mid‐gestation (Rands, 1986b; Snow & Tam, 1979; Tarkowski, 1959).

Besides these differences in regulation of the overall embryo size at gastrulation, spinal cord formation also differs between species (Leung & Shimeld, 2019; Steventon & Martinez Arias, 2017). One key difference is in the pool of NMP cells that are incorporated into the spinal cord at the onset of its formation. In fast developing species, such as zebrafish, limited pools of nonproliferating progenitors are thought to generate specific AP regions of the neural tube (Attardi et al., 2018; Steventon et al., 2016). By contrast, in mice NMPs persist over at least 2 days of development and their pool is replenished by cell proliferation (Cambray & Wilson, 2002; Tzouanacou, Wegener, Wymeersch, Wilson, & Nicolas, 2009), although precise quantitative estimates of the number of NMPs are currently lacking. These differences are consistent with the much larger volumetric growth of the posterior body observed in mice compared to zebrafish (Steventon et al., 2016). It is conceivable that the proliferative capacity of NMPs in mammals enables them to compensate for any cell loss and achieve a defined number of progenitors that will give rise to a spinal cord of specific size. Alternatively, the size and robustness of patterning of the spinal cord in mammals might rely on the regulation of tissue size prior to neural tube formation.

To get an insight into how much the size of the NMP pool influences subsequent spinal cord development, it will be necessary to get quantitative estimates of the size of this cell population in normal development, as well as in conditions in which it is specifically perturbed. For instance, in Tbx6 and Wnt3a mouse mutants the balance between neural and mesodermal descendants is altered, leading to the specification of more Sox2 positive neural progenitors at the expense of paraxial mesoderm. Interestingly, rather than having a larger neural tube, these mutants have several small ectopic neural tubes. This indicates that the specification of cell identity is decoupled from the movement of cells from the CLE to the prospective neural plate and paraxial mesoderm regions (Takada et al., 1994; Takemoto et al., 2011; Yoshikawa, Fujimori, McMahon, & Takada, 1997). Therefore, the size of the spinal cord is likely to depend not only on the number of available NMPs, but also on the morphogenetic movements involved in neural plate formation.

## 3 CELL CYCLE PROGRESSION AND THE CONTROL OF CELL NUMBER

### 3.1 Proliferation rate

Neural progenitors within the spinal cord are highly proliferative. Across the DV length of the neural tube, the proliferation rate of progenitors is spatially uniform and independent of progenitor identity (Kicheva et al., 2014). Similar to many developing tissues (Lange & Calegari, 2010), the proliferation rate of neural progenitors decreases and their cell cycle lengthens as developmental time progresses (Kauffman, 1968; Kicheva et al., 2014; Wilcock et al., 2007), (Table 1). Because the anterior neural tube is developmentally older than the posterior, this decrease in the proliferation rate is reflected along the anterior–posterior axis, with the highest proliferation rates observed at the posterior end (Olivera‐Martinez et al., 2014).

**TABLE 1:** Growth parameters of the mouse neural tube

		E8	E8.5	E9.5	E10.5	E11.5
DV length (μm)		99	155	326	420	477
AB length (μm)	pD	32	45	78	98	102
	pMN	24	34	56	35	32
Number of progenitors		75	126	360	599	834
Cell cycle length (hr)	pD	8	8	9	12	14
Differentiation rate (1/hr)	pD	0	0	0.02	0.03^a^	0.01^b^
	pMN	0	0	0.11	0.14^a^	0.11^b^
Apoptotic index (1 × 10^−3^)	pD	4	2	2	3	0
		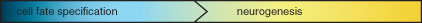				

*Note*: Mean values of the parameters indicated in the first column at developmental stages from E8 to E11.5, data from Kicheva et al. (2014). All parameters were quantified from brachial‐level transverse sections. Number of progenitors represents Sox2+ cells per hemisection (left or right side of the neural tube). Cell cycle length was estimated from the fraction of pH3+ progenitors (mitotic index) and the measured length of mitosis. Differentiation rate is defined as the fraction of progenitors that become postmitotic neurons per hour. The mean number of progenitors and mean cumulative number of neurons at different stages were used for calculation. Superscripts: a, measured at E10; b, measured at E11. Apoptotic index was measured as the fraction of progenitors positive for cleaved Caspase 3.

While cell proliferation is key for generating tissue mass by exponential increase in cell number, it is also inherently sensitive to noise (Lander, 2011), and if left uncontrolled, can cause strong disturbances in tissue size (Copp & Greene, 2010; Zechner et al., 2003). Because of this, the proliferation rate is under tight regulation and multiple cell‐intrinsic and ‐extrinsic mechanisms are likely to contribute to this control. These regulatory mechanisms have to be consistent with the observed spatiotemporal dynamics of the rate of proliferation at the tissue level.

Some of the best‐known extrinsic regulators of proliferation in the spinal cord are the morphogens that also control pattern formation (Ulloa & Briscoe, 2007). Fgf8 signaling in the caudal neural plate maintains progenitors in a proliferative state (Diez del Corral, Breitkreuz, & Storey, 2002) and favors symmetric self‐expanding divisions (Wilcock et al., 2007). Fgf signaling is inhibited by RA secreted by the somites (Diez del Corral et al., 2002), hence neural progenitors are exposed to it only transiently at early stages of spinal cord formation when their proliferation rate is high. Shh and Wnt signaling have also been proposed to promote neural progenitor proliferation. A number of studies have shown that abrupt gain of Shh or Wnt function results in increased progenitor proliferation, decreased neuronal differentiation and overgrowth of the spinal cord (Alvarez‐Medina, Le Dreau, Ros, & Martí, 2009; Cayuso, Ulloa, Cox, Briscoe, & Marti, 2006; Chesnutt, Burrus, Brown, & Niswander, 2004; Dickinson, Krumlauf, & McMahon, 1994; Megason & McMahon, 2002; Rowitch et al., 1999; Zechner et al., 2003). By contrast, the spinal cord is smaller upon β‐catenin deletion, loss of Shh or Wnt1 and Wnt3a (Chiang et al., 1996; Ikeya, Lee, Johnson, McMahon, & Takada, 1997; Muroyama, Fujihara, Ikeya, Kondoh, & Takada, 2002; Zechner et al., 2003).

While these data suggest that Fgf, Shh, and Wnts promote proliferation, several factors pose a challenge in interpreting how exactly these signaling pathways act. On one hand, it is difficult to distinguish whether these pathways regulate the probability of progenitors to differentiate into postmitotic neurons or change the length of the cell cycle. This is because the probability to differentiate is often correlated with the length of the cell cycle or a specific cell cycle phase (Arai et al., 2011; Calegari, Haubensak, Haffher, & Huttner, 2005; Peco et al., 2012; Wilcock et al., 2007). Consistent with this observation, symmetric divisions that generate two progenitors (PP divisions) have been associated with shorter cell cycle length than asymmetric divisions, which generate a progenitor and a neuron (PN divisions) (Wilcock et al., 2007). Thus, a bias from self‐renewing PP divisions to asymmetric neuron‐generating PN divisions could simultaneously result in increasing the probability to differentiate and decreasing the overall rate of progenitor proliferation. Activation of Fgf, Shh, and Smad1/5 signaling has been reported to increase the frequency of self‐renewing PP divisions at the expense of neuron‐generating divisions, suggesting that these pathways alter the probability to differentiate (Le Dréau, Saade, Gutiérrez‐Vallejo, & Martí, 2014; Saade et al., 2013; Saade, Gonzalez‐Gobartt, Escalona, Usieto, & Martí, 2017; Wilcock et al., 2007). Whether these signaling pathways also directly alter cell cycle length is still poorly understood.

**FIGURE 3:**
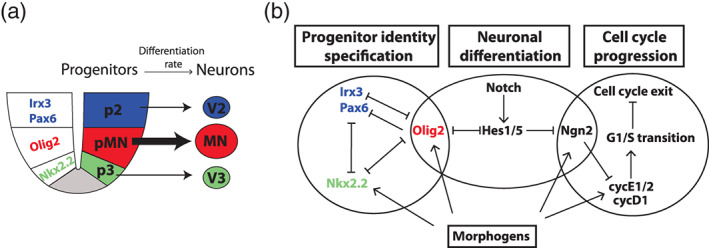
Gene regulatory networks controlling progenitor specification, neuronal differentiation and cell cycle progression in the spinal cord. (a) The identities of p3, pMN, and p2 progenitors in the ventral neural tube are regulated by the expression of transcription factors, such as Nkx2.2, Olig2, Pax6, and Irx3. Starting from ~E9 of mouse development, the pMN progenitors differentiate at a higher rate than progenitors from p2 and p3 domains (Kicheva et al., 2014). (b) A simplified diagram of the interactions between the gene regulatory networks involved in cell fate specification, neuronal differentiation and cell cycle regulation. Shared genes from these GRNs are key in coordinating the specification of progenitor identities, the transition from progenitors to postmitotic neurons, and the cell cycle dynamics (Lacomme, Liaubet, Pituello, & Bel‐Vialar, 2012; Lobjois et al., 2008; Sagner et al., 2018). Components of these GRNs are controlled by the extrinsic factors, such as morphogens. Many components and interactions are omitted

To address this question, it would be necessary to gain a better understanding of how differentiation, mode of division, and cell cycle progression are linked. In the spinal cord neurons are generated both by symmetric (NN) and asymmetric (PN) divisions, hence there is no strict association between the overall rate of differentiation and the mode of division (Morin, Jaouen, & Durbec, 2007; Wilcock et al., 2007). Randomization of the spindle orientation does not change the frequency of PP, PN and NN divisions suggesting that fate acquisition is controlled independently of spindle orientation (Morin et al., 2007). Furthermore, cell cycle length and neuronal differentiation can be experimentally uncoupled. For instance, overexpression of D‐type cyclins leads to the production of cells that express markers of mature neurons, but continue to proliferate outside of the progenitor zone (Lobjois, Bel‐Vialar, Trousse, & Pituello, 2008). Expression of a mutant variant of Cdc25B, which is unable to interact with cyclin‐dependent kinase 1, can alter the proportion of neurogenic divisions without affecting the duration of the G2 phase (Bonnet et al., 2018). These observations suggest that cell cycle and neuronal differentiation are controlled by largely separate molecular mechanisms (Figure 3). These molecular regulatory modules have shared components, yet how exactly the coordination between them is achieved in the spinal cord is a key outstanding question (Azaïs et al., 2019; Bonnet et al., 2018), see also Section 3.2 and (Hardwick, Ali, Azzarelli, & Philpott, 2015).

To understand the specific effect of morphogens on the cell cycle, it will be useful to obtain a better understanding of the components within the core cell cycle machinery that they control. The expression levels of N‐Myc and D‐type cyclins frequently correlate with changes in the rate of proliferation, and are also affected by perturbations in Shh, Wnt, and Fgf signaling (Alvarez‐Medina et al., 2009; Cao, Pfaff, & Gage, 2008; Cayuso et al., 2006; Lobjois, Benazeraf, Bertrand, Medevielle, & Pituello, 2004; Lukaszewicz & Anderson, 2011; Megason & McMahon, 2002). Cyclins D1 and D2 are expressed in spatially distinct domains along the DV axis and play a role in cell fate specification (Lobjois et al., 2004; Lukaszewicz & Anderson, 2011; Olivera‐Martinez et al., 2014). However, whether the expression levels of D‐type cyclins are sufficient to explain changes in the proliferation rate is poorly understood.

Another major challenge is understanding how spatially uniform proliferation can be regulated by graded morphogen signaling along the DV axis. If the signaling levels of a given morphogen pathway directly controlled the rate of proliferation, this would imply that the rate of proliferation should also be graded in space. This is contrary to experimental observations (Kicheva et al., 2014), thereby ruling out a scenario in which the absolute level of signaling of a particular morphogen pathway is proportional to the rate of proliferation. An analogous problem exists in the *Drosophila* wing disc. Multiple models have been proposed to explain how uniform proliferation might be regulated in this tissue (reviewed in Dekanty & Milán, 2011; Schwank & Basler, 2010; Wartlick, Mumcu, Jülicher, & Gonzalez‐Gaitan, 2011). For instance, cell division could be controlled by the relative increase in BMP signaling levels over time (Wartlick, Mumcu, Kicheva, et al., 2011). Another possibility is that morphogens might be simply permissive for proliferation above a certain threshold, while other signals or mechanical forces would determine the cessation of growth (Aegerter‐Wilmsen, Aegerter, Hafen, & Basler, 2007; Hufnagel, Teleman, Rouault, Cohen, & Shraiman, 2007). Whether similar mechanisms operate in the spinal cord is unknown.

The regulation of cell proliferation is fundamental to spinal cord development, yet many questions from tissue to molecular level remain open. Tissue growth and morphogen gradient formation are inherently linked processes (Averbukh, Ben‐Zvi, Mishra, & Barkai, 2014; Fried & Iber, 2014; Wartlick, Mumcu, Kicheva, et al., 2011), and the feedbacks between them can reveal properties that are crucial for development (Aguilar‐Hidalgo et al., 2018). Furthermore, multiple signaling pathways and intrinsic mechanisms contribute to regulating the cell cycle, but how they are balanced to achieve proliferation that is uniform in space and declining in time is unclear. It also remains to be determined how signaling pathways and other regulatory inputs target the cell cycle machinery to exert their effects. Advanced live imaging techniques, ex vivo systems, and new genomic approaches combined with a quantitative understanding of cell cycle kinetics offer an excellent opportunity to address these questions.

### 3.2 Terminal differentiation

Once subtype identities are established, progenitors begin to differentiate into postmitotic neurons of the respective type. Thus, the molecular transition from progenitor to neuronal identity is accompanied by cell cycle exit. Although the two processes are controlled by largely separable mechanisms, they are coupled by factors such as Ngn2 (Figure 3) (Lacomme et al., 2012; Lobjois et al., 2008). Neurons are transiently attached to the apical side of the tissue via a thin process with severely reduced apical surface, and are subsequently lost from the neuroepithelium via apical abscission (Baek et al., 2018; Das & Storey, 2014). Thus, the rate of differentiation reflects the rate of progenitor loss and it varies between progenitor domains and in time (Table 1). Because the proliferation rate is spatially uniform, the sizes of progenitor domains during neurogenesis are determined by the cell type‐specific differentiation rates (Kicheva et al., 2014). In this way, terminal differentiation affects not only the overall size of the neural tube, but also the progenitor pattern and the number of postmitotic neurons.

The observation that progenitor types differ in their rate of differentiation suggests that cell intrinsic mechanisms regulate neuronal differentiation. This process is indeed dependent on proneural genes, such as Ngn2 and Ascl1, as well as Notch ligands, which are expressed in a domain‐specific manner (Gowan et al., 2001; Marklund et al., 2010). These genes interact with the gene regulatory network that controls progenitor identity (Figure 3). One of the best understood examples is the regulation of the differentiation rate of pMN progenitors into motor neurons. The transcription factor Olig2 is expressed in response to Shh and RA and is required to confer pMN identity (Briscoe & Ericson, 2001; Novitch, Chen, & Jessell, 2001; Novitch, Wichterle, Jessell, & Sockanathan, 2003; Takebayashi, Nabeshima, Yoshida, Chisaka, & Ikenaka, 2002). Olig2 inhibits and is itself inhibited by Hes1 and Hes5, effectors of the Notch pathway that maintain neural progenitors in a proliferative undifferentiated state (Sagner et al., 2018). By a yet unknown mechanism, the levels of Olig2 increase over time, which leads to the repression of Hes1/5. This in turn allows enhanced expression of the proneural gene Ngn2, which promotes motor neuron generation (Sagner et al., 2018). This illustrates how a cell identity gene (Olig2), by interacting with the regulatory network that executes the transition from progenitors to postmitotic neurons, can provide cell type‐ specific control of differentiation (Figure 3).

Whether the temporal profile of terminal differentiation can be explained entirely by a cell intrinsic mechanism, in which the dynamics of the gene regulatory network contains an inherent cell‐autonomous switch from progenitor to neuron stage, remains an open question. It has been observed that in proliferating cortical progenitors the expression of bHLH factors such as Hes1/5 and Ngn2 oscillates, whereas at the onset of differentiation it becomes stable (Imayoshi et al., 2013; Shimojo, Ohtsuka, & Kageyama, 2008). Although this might differ in the spinal cord, it is possible that temporal change in some factor that interacts with the proneural gene network, such as Olig2 (Sagner et al., 2018), mir9 (Bonev, Stanley, & Papalopulu, 2012), or Ngn2 itself (Ali et al., 2011), drives the transition to stable proneural gene expression. In support of this view, motor neuron generation in vitro follows similar species‐specific kinetics to in vivo (reviewed in Ebisuya & Briscoe, 2018). Further work is required to understand how the temporal changes in differentiation regulators are brought about. Live imaging of differentiation regulators in individual cells together with mathematical modeling has been exploited in recent years as a powerful approach for studying the differentiation kinetics (Imayoshi et al., 2013; Manning et al., 2019; Sagner et al., 2018). Expanding on these studies to assess the contribution of different inputs to differentiation during normal development in individual cells will help to establish whether intrinsic mechanisms are sufficient to account for the dynamics of differentiation rates in the neural tube.

Extrinsic factors, such as morphogens, can also contribute to the temporal dynamics of differentiation. Both Shh and BMP have been suggested to bias the cell division mode and in this way alter the balance between progenitor and neuron generation (Le Dréau et al., 2014; Saade et al., 2013, 2017). The signaling levels of both of these pathways decline over time (Kicheva et al., 2014; Zagorski et al., 2017). Thus, high levels of Shh signaling were suggested to promote proliferative and prevent neurogenic divisions at early stages (Saade et al., 2013). A challenge in understanding the contribution of the temporal dynamics of the Shh signaling pathway to neurogenesis is to reconcile this type of model with the distinct rates of differentiation observed in different progenitor domains. Furthermore, it will be important to distinguish possible indirect effects of signaling pathways on progenitor identity from their influence on the mode of cell division.

Much progress has been made in uncovering the mechanisms that drive terminal differentiation, however, many questions remain open. The role of extrinsic signals and the influence of mechanical forces in this process (Hiscock, Miesfeld, Mosaliganti, Link, & Megason, 2018; Streichan, Hoerner, Schneidt, Holzer, & Hufnagel, 2014) have not been systematically studied. Furthermore, it is unclear how the cellular and molecular mechanisms that underlie neuronal differentiation relate to the functional requirements of the spinal cord. From a functional point of view, what is relevant for the animal is robust formation of neural circuits that are adapted to the size of the target tissue that they need to innervate. To achieve this adaptation, postmitotic neurons compete for neurotrophic factors released by the targets, which causes the elimination of a significant proportion of neurons (Huang & Reichardt, 2001; Raff et al., 1993). This makes it challenging to understand how the number of progenitors and their relative proportions in the embryonic spinal cord relate to the construction and functionality of the final neural circuits. To make progress in this direction, it will be necessary to investigate potential feedbacks between proliferating progenitors and differentiated neurons, both surviving and undergoing cell death (Agasse, Roger, & Coronas, 2004; Lander, Gokoffski, Wan, Nie, & Calof, 2009). Obtaining quantitative measurements of the neuron pools and an overall quantitative understanding of the process will be key in this undertaking.

### 3.3 Apoptosis

Some tissues, such as the mouse epiblast (Clavería et al., 2013; Sancho et al., 2013), *Drosophila* embryonic segments (Crossman, Streichan, & Vincent, 2018; Parker, 2006), and neuroblasts (Bello, Hirth, & Gould, 2003; reviewed in Pinto‐Teixeira, Konstantinides, & Desplan, 2016) eliminate cells through apoptosis during their normal development. This process can play different roles, for instance remove defective cells (M. Merino et al., 2015), help sculpt tissues (R. Merino et al., 1999), or relieve mechanical pressure (Levayer, Dupont, & Moreno, 2016). Apoptosis has also been suggested to regulate tissue size (Crossman et al., 2018) and correct pattern by eliminating mis‐specified cells (Hashimoto & Sasaki, 2019; Morris et al., 2012) or shaping the morphogen‐producing domain (Nonomura et al., 2013).

Consistent with a link between pattern formation and cell survival, apoptosis frequently occurs in tissues where patterning cues, such as morphogens or transcription factors that confer cell identity, are defective (Adachi‐Yamada, Fujimura‐Kamada, Nishida, & Matsumoto, 1999; Crossman et al., 2018; Litingtung & Chiang, 2000). This is also the case in the spinal cord. In wildtype mouse embryos during the first days of spinal cord development, apoptosis is observed at very low levels (Kicheva et al., 2014; Table 1), except at the dorsal boundary of the neuroepithelium upon neural tube closure (Massa et al., 2009; Yamaguchi et al., 2011). However, in embryos with Shh loss of function, neural progenitors undergo substantial cell death at E9.5 of development (Litingtung & Chiang, 2000). Cell death in Shh null mutants is rescued by the removal of the downstream repressor of Shh signaling—Gli3 (Cayuso et al., 2006; Litingtung & Chiang, 2000), indicating that Shh transcriptionally regulates survival factors. Consistent with this, the anti‐apoptotic factor Blc‐2 has been shown to be a direct target of Shh signaling (Peterson et al., 2012). In addition, in Shh Gli3 double mutants, the Shh receptor Ptch1 is expressed at high levels (Litingtung & Chiang, 2000), indicating that unliganded Ptch1 does not directly induce apoptosis as previously suggested (Thibert et al., 2003). Further investigation of Gli3 transcriptional targets as well as characterization of the spatiotemporal profile of cell death in conditions where Shh signaling is altered are necessary to establish how cells interpret Shh signaling to control their survival.

Other morphogen pathways might also be involved in the regulation of cell survival in the neural tube. Analysis of constitutive mouse mutants for the BMP inhibitors Noggin and Chordin suggests that overactivation of BMP signaling promotes apoptosis in the dorsal neural tube (Anderson, Stottmann, Choi, & Klingensmith, 2006; McMahon et al., 1998). However, in these mutants the observed phenotypes might result from a disruption of an earlier function of BMP signaling in development. Acute overexpression of constitutively active BMPR1b in chick induces apoptosis at early stages (HH10‐12), and this effect is recapitulated by overactivation of the BMP downstream target gene Msx1 (Liu, Helms, & Johnson, 2004). By contrast, loss of BMP signaling in BMPR1a, BMPR1b double mutant mouse embryos does not affect progenitor survival and neural tube growth (Wine‐Lee et al., 2002). Although more research is needed to understand the role of BMP signaling in cell survival, these results suggest that BMP signaling might not directly control the apoptosis machinery, but some patterning errors resulting from perturbations in BMP signaling might induce cell death.

Strong perturbations in morphogen signaling are often fatal for embryonic development and it is difficult to discern to what extent it is the changes in tissue size or patterning that contribute to this outcome. Nevertheless, in the spinal cord of Minute (Bst/+) mice, increased cell death starting at E9.0 results in a smaller neural tube with scaled pattern (Kicheva et al., 2014). These mice survive to adulthood with a ~20% decrease in body size and minor retinal and skeletal defects (Oliver, Saunders, Tarlé, & Glaser, 2004; Tang, Rice, & Goldowitz, 1999), suggesting that animals can cope with a certain overall reduction in spinal cord size as long as pattern scaling is maintained. This raises the possibility that position‐dependent control of apoptosis downstream of morphogens and patterning genes might function to restore defects in pattern at the expense of some reduction in neural tube size. Quantitative analysis of cell survival in conditions where morphogen signaling levels can be precisely manipulated is required to address this possibility.

## 4 CELL SIZE AND SHAPE

In the first three days of spinal cord formation in mouse, the number of progenitors and neurons increases by approximately an order of magnitude (Table 1; Kicheva et al., 2014) and this increase in cell number is the main factor that leads to neural tube growth. At the same time, neuroepithelial cells also undergo significant changes in size and shape. These changes are reflected in the degree of pseudostratification of the neuroepithelium. At the earliest stages of spinal cord development, the neural plate of amniotes is a thin epithelium with two to three layers of nuclei. With time, increasing layering of the nuclei leads to a two to threefold increase in the AB thickness of the neuroepithelium (Table 1) and denser packing of the nuclei within the progenitor layer (Ferreira, Despin‐Guitard, Duarte, Degond, & Theveneau, 2019; Kicheva et al., 2014; Schoenwolf & Powers, 1987; Schoenwolf & Smith, 1990). When neuronal differentiation commences and mature neurons delaminate from the progenitor layer into the mantle zone, the AB length of the epithelium begins to decrease. The rate of neuronal differentiation varies between DV domains, and accordingly, the epithelial AB thickness varies along the DV axis during neurogenesis.

These changes along the AB axis of progenitors are accompanied by changes of progenitor shape at the apical surface of the neural tube. This is most obvious when comparing early neural plate to later developmental stages. With time, the apical surfaces of neural progenitors become more regularly packed, with smaller variation in cell area (Escudero et al., 2011; Grego‐Bessa et al., 2016). At E11.5 of mouse development, the apical area of progenitors at the brachial level corresponds to ~8 μm^2^, and, similar to other epithelia, cells have on average 5.8 neighbors (Guerrero et al., 2019). Nevertheless, systematic measurements of neural progenitor cell shapes throughout the specification and neurogenesis stages are still lacking. Similarly, changes in cell volume have been inferred from bulk tissue measurements (Schoenwolf & Powers, 1987), however, detailed reconstructions at the single cell level will be necessary to uncover the discrete dynamics of cellular growth.

How the temporal regulation of cell shape is established is poorly understood. Many studies have focused on the morphogenetic and cellular changes within the neural plate leading to neural tube closure (for review see Nikolopoulou, Galea, Rolo, Greene, & Copp, 2017). During this process, multiple mechanisms, such as cell intercalation, convergent extension and apical constriction, are dynamically coordinated by Wnt/planar cell polarity (PCP) signaling (Nishimura, Honda, & Takeichi, 2012; Williams, Yen, Lu, & Sutherland, 2014; Ybot‐Gonzalez, Savery, et al., 2007), mechanical forces (Galea et al., 2017), as well as BMP antagonism (Ybot‐Gonzalez, Gaston‐Massuet, et al., 2007). However, the factors that contribute to increasing pseudostratification and modifications of cell shapes later in development are poorly understood.

On one hand, the changes in the AB thickness of the epithelium may result directly from alterations in the cell cycle length and the rate of neuronal differentiation. Consistent with this, the cell cycle regulator CDK1 is linked with the cytoskeleton and interkinetic nuclear movements (Strzyz et al., 2015), hence it is possible that the rate of proliferation and cell shape are intrinsically coupled by common regulators. Furthermore, under normal conditions neuronal differentiation coincides with a decrease in the AB length of the progenitor layer and forcing neural progenitors to differentiate prematurely has a similar effect (Herrera, Saade, Menendez, Marti, & Pons, 2014; Kicheva et al., 2014). These observations suggest that neuronal differentiation also affects cell shape.

Cell cycle independent mechanisms might also contribute to the regulation of cell shape. In the anterior neural plate, loss of PTEN activity impairs the ability of the neuroepithelium to increase its AB thickness—an effect that was independent of cell proliferation and suggested to depend on apical‐to‐basal vesicle trafficking (Grego‐Bessa et al., 2016). Mechanical forces can also contribute to epithelial packing (Guerrero et al., 2019; Hiscock et al., 2018; Streichan et al., 2014). Altogether, further studies are necessary to determine how the AB length is regulated over time in the spinal cord.

## 5 TISSUE SHAPE ANISOTROPY

The spinal cord has a remarkably anisotropic shape—it spans the body axis in AP direction, while its DV and AB dimensions are much smaller. Much of the AP length of the neural tube arises from the progressive extension at the posterior end, which results from the addition of neural progenitors from the NMP pool and from the high proliferation rate of newly generated neural progenitors. In addition, the neural plate undergoes convergent extension, which elongates the tissue in the AP direction (Steventon et al., 2016). This process is controlled by PCP signaling and mutations in this pathway lead to truncation of the body axis (reviewed in Nikolopoulou et al., 2017).

As the spinal cord extends along the AP axis, the adjacent somitic mesoderm, notochord, endoderm, and surface ectoderm are also elongating in order to reach equivalent AP lengths. However, all these tissues proliferate and grow in volume at different rates (Steventon et al., 2016). Furthermore, cells within these tissues can undergo some degree of directed motion with properties (speed, coherence) that vary with distance to the posterior end of the embryo (Bénazéraf et al., 2010; Lawton et al., 2013; Sausedo & Schoenwolf, 1994; Xiong, Ma, Benazeraf, Mahadevan, & Pourquie, 2018). Together, these processes result in complex morphogenetic movements and sliding of the tissue layers with respect to each other as they extend along the AP axis. Although it has been suggested that these tissues have independent intrinsic size regulation (Snow et al., 1981), mechanical and biochemical feedbacks between them might contribute to coordinating their AP extension (Mongera, Michaut, Guillot, Xiong, & Pourquié, 2019). Still, how the overall length of the spinal cord and adjacent tissues is achieved in a coordinated manner is a key unresolved question.

While convergent extension contributes to extension of the neural tube in AP direction at early stages, at later stages (E11.5 of mouse development) the neural tube extends overall more in DV than in AP direction (Kicheva et al., 2014). This is reflected by the dorsoventrally elongated shapes of lineage‐traced cell clones in most of the progenitor domains. The only exception is the motor neuron domain, where the clone shapes are isotropic, indicating equal growth along the DV and AP axes. A computational vertex model of the mouse neuroepithelium revealed that the higher rate of progenitor loss due to terminal differentiation in the motor neuron progenitor domain is sufficient to explain this distinct decrease in growth anisotropy (Guerrero et al., 2019). Thus, under global anisotropic mechanical constraints, the growth rate determines how much faster the tissue elongates in DV than in AP direction. While it is unclear how these global mechanical forces are generated, this shows that the anisotropy of growth does not depend on oriented cell divisions, which are random in the plane of the epithelium at these stages, or on domain‐specific directional cues.

Many questions about the regulation of directional growth remain open. A major challenge is to develop assays that would allow direct measurements of the mechanical properties of the neural epithelium at different developmental stages. A better understanding of the feedbacks between mechanical influences and the rates of proliferation, differentiation and apoptosis are also necessary. In addition, gaining insight into the relationships between neighboring tissues, both at biochemical and mechanical level, will be a crucial component of understanding how the shape of the spinal cord is generated and elaborated over time.

## 6 INTEGRATED REGULATION OF SPINAL CORD SIZE

### 6.1 Is there a size‐sensing mechanism?

When transplanted into the abdomen of a fly, a *Drosophila* wing imaginal disc grows to its appropriate size (Bryant & Levinson, 1985). This and other experiments illustrated existence of a disc‐autonomous size control mechanism that allows the tissue to grow to a predetermined final size. A later study demonstrated that imaginal discs measure absolute tissue dimensions, rather than cell number or cell size (Neufeld, De La Cruz, Johnston, & Edgar, 1998). The organ intrinsic growth program in the wing disc implies that size is sensed in some way. Thus, perturbations in growth that do not impair this sensing mechanism can be corrected.

In the spinal cord, the question of whether there is a size‐sensing mechanism is largely unresolved. In mice, experimental conditions such as Bst/+, in which a fraction of the cells is eliminated by apoptosis, yield smaller animals with fewer progenitor cells in their spinal cords during development (Kicheva et al., 2014). In zebrafish, surgical removal of a fraction of the cells prior to gastrulation produces smaller neural tubes (Collins et al., 2018). These observations suggest that the spinal cord might be able to sustain large variations in size and that alterations in cell number are not necessarily repaired. On the other hand, compensatory proliferation has been reported to occur at early stages of spinal cord formation in several mammalian species (Snow et al., 1981; Snow & Tam, 1979). Furthermore, ablation of a hemi‐segment of the chick neural tube up to stage HH17 leads to complete regeneration of the missing tissue (Halasi, Søviknes, Sigurjonsson, & Glover, 2012). These experiments imply that the neural tube possesses some capacity to regulate its size, at least at early developmental stages. Yet, the current state of knowledge reflects a need for a better understanding of the overall phenomenology of size control in the spinal cord. In this effort, differences between species will be an important consideration, since the growth properties of the spinal cord differ between species (Steventon & Martinez Arias, 2017).

The mechanisms by which size might be sensed and regulated are also poorly understood. One possibility is that it is accomplished via global tissue‐scale regulators, such as a system of opposing morphogen gradients (reviewed in Briscoe & Small, 2015; Shilo & Barkai, 2017) or mechanical properties (Irvine & Shraiman, 2017). An alternative is that size is an emergent property, resulting from local regulation at the level of individual cells or subsets of cells. Such local regulation could entail, for instance, feedbacks between specific subtypes of neurons and the differentiation rate of respective progenitors (Lander et al., 2009), or modification of the terminal differentiation rate as a result of local cell crowding (Hiscock et al., 2018). Future studies are needed to establish whether and how such local feedbacks operate in the spinal cord, and how they might be coordinated with global mechanisms of tissue size regulation.

### 6.2 Scaling of pattern with tissue size

The DV pattern of the neural tube is highly dynamic during development (Balaskas et al., 2012; Delile et al., 2019; Ericson, Briscoe, Rashbass, Van Heyningen, & Jessell, 1997; Junker et al., 2014; Kicheva et al., 2014). At the beginning of spinal cord formation in mouse, the dynamics of morphogen signaling gradients and cross‐interactions between target genes lead to changes in cell identities that shift the boundaries of gene expression domains. After the onset of neurogenesis, the boundary positions of progenitor domains continue to shift due to domain‐specific rates of terminal differentiation. The consequence of this dynamics is that pattern does not scale with the growing tissue size in the course of development, that is, the sizes of progenitor domains do not change in proportion with the tissue size over time (Kicheva & Briscoe, 2015). Nevertheless, the question of pattern scaling arises in the context of comparing individuals from the same species with different overall size, as well as in the context of inter‐species comparisons.

In different species, marked differences in DV length at equivalent AP positions are observed soon after the onset of spinal cord development. For instance, two days after headfold stage, the DV length of the chick and zebrafinch neural tube differs by approximately twofold (Uygur et al., 2016). Despite differences in overall size, the progenitor pattern is remarkably conserved across vertebrate species (Leung & Shimeld, 2019). Although quantitative comparisons between species are rarely available, comparisons between mouse and chick (Kicheva et al., 2014) revealed that the proportions of different progenitor domains are to a large degree preserved in these species across developmental time. Similarly, pattern scales between chick and zebrafinch, albeit it proceeds faster in the zebrafinch (Uygur et al., 2016). Pattern scaling in the neural tube has also been observed within the same species, in cases where tissue size was perturbed. In Minute heterozygous mutant mice, which exhibit excessive cell death and a significantly smaller neural tube size from E9 onwards, the pattern of progenitor domains scales with the wildtype throughout developmental time (Kicheva et al., 2014). In surgically reduced zebrafish embryos, pattern scaling has also been observed (Collins et al., 2018).

**FIGURE 4:**
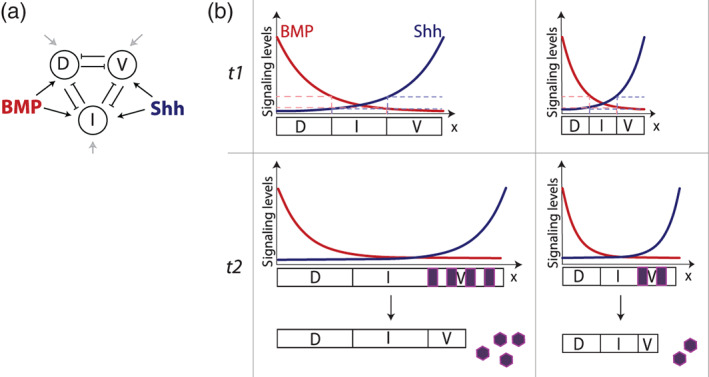
Scaling of pattern with tissue size. (a) A simplified morphogen‐controlled gene regulatory network in the spinal cord. Shh and BMP signals activate expression of dorsal (D), intermediate (I) and ventral (V) target genes, which repress each other. Input from uniformly expressed activators is marked with gray arrows. This system results in the formation of three adjacent domains of D, I, V expression as shown in (b). (b) A possible model of growth and pattern for two individuals that have tissues with different initial sizes. In both individuals throughout the initial specification phase of development (t1), the opposing gradients have decay lengths that scale with tissue size and identical amplitudes. The morphogen‐driven network in (a) leads to the formation of dorsal (D), intermediate (I) and ventral (V) domains along the DV axis. The levels of morphogen signaling at the boundaries between domains in both cases are the same. During the neurogenesis phase (t2), the tissues have expanded in DV length by uniform proliferation, the levels of signaling of both morphogens have decreased. The pattern is maintained by the downstream gene regulatory network (a). Progenitors in the V domain begin differentiating into postmitotic neurons at a rate independent of tissue size, allowing for progenitor domain proportions to be maintained between individuals

These examples of pattern scaling between individuals raise the question of how scaling is achieved. Studies in *Drosophila*, as well as DV patterning of the early amphibian embryo, have drawn attention to the scaling of morphogen gradients with tissue size (Ben‐Zvi, Pyrowolakis, Barkai, & Shilo, 2011; Inomata, Shibata, Haraguchi, & Sasai, 2013; Wartlick, Mumcu, Kicheva, et al., 2011). Mechanisms that rely on an expander that regulates the morphogen range, opposing morphogen gradients, and others have been proposed to explain gradient scaling to tissue size (Kicheva & Briscoe, 2015; Shilo & Barkai, 2017; Wartlick, Mumcu, Jülicher, et al., 2011). In the spinal cord, a model for the establishment of pattern in response to opposing signaling gradients of BMP and Shh (Figure 4a) implies that pattern would scale as long as the amplitudes and the relative decay lengths of the signaling activity gradients are constant for different tissue sizes at equivalent developmental time points (Zagorski et al., 2017) (Figure 4b). Consistent with this, scaling of the expression profile of a reporter for Ptch, a direct Shh target gene, has been observed in surgically reduced zebrafish embryos at 20 hpf (Collins et al., 2018). In zebrafinch and chick, the Shh activity profiles are not known, but the Shh gradient has a lower amplitude and the target cells are more sensitive to Shh in the zebrafinch, suggesting that scaling is achieved by alterations in both the ligand profile and signal transduction cascade (Uygur et al., 2016).

The nonlinear relationship between ligand and signal, and the temporal dynamics of the signaling profiles, pose a challenge in understanding how scaling at the level of signaling activity is achieved. In the case of Shh, the ligand gradient increases in amplitude and maintains a constant decay length during development, while at the same time the signaling activity declines due to temporal adaptation (Cohen et al., 2015). Much work is needed, on one hand, to collect quantitative data of ligand, signaling activity and downstream gene expression profiles in different model systems, and on the other, to develop theoretical frameworks that allow us to better understand the logic that ensures scaling in such nonlinear signaling systems.

Even though scaling of the morphogen signaling profiles could explain the scaling of pattern upon its early specification, other mechanisms must account for pattern scaling during the subsequent phase of neurogenesis and anisotropic growth (Figure 4b). This is especially obvious in mammals, where the regulation of size at gastrulation (as discussed earlier) results in highly reproducible tissue size during the early phase of pattern formation, but later in development tissue growth may differ substantially. Progenitor pattern scaling between mouse and chick, as well as between wildtype and Minute mouse embryos, has been observed during the neurogenesis phase, after the initial pattern is established in response to morphogen signaling (Kicheva et al., 2014). For this to be achieved, the difference between the differentiation rate of distinct progenitor domains over time has to be independent of the overall tissue size in later phases of development (Kicheva et al., 2014), (Figure 4b). This is consistent with the observed differentiation rate dynamics in mouse, chick and *Minute* mouse mutants (Kicheva et al., 2014). Such dynamics could be explained by a mechanism in which the rate of differentiation is determined by a cell type‐specific gene regulatory network that involves the cell identity genes (Sagner et al., 2018), rather than a global size‐dependent cue. Further studies of the regulation of the rate of differentiation will help to shed light on how scaling might be achieved during neurogenesis.

In addition to the spatial growth anisotropy created by unequal rates of neuronal differentiation, the temporal changes in the rate of cell loss due to differentiation have a strong impact on the progenitor pattern. While the timescale of developmental events happens to be similar between mouse and chick, it differs significantly between other vertebrate species. The peak rate of motor neuron generation, for instance, shifts in time according to the overall developmental pace (reviewed in Ebisuya & Briscoe, 2018). Understanding the mechanisms that account for these temporal differences in cell cycle progression rates between species will help shed light on how the temporal and spatial aspects of pattern scaling are coordinated.

The observation that the neural progenitor pattern scales between individuals, at least in some cases, raises the question of the functional relevance of this scaling. Scaling implies that the absolute number of progenitors changes between individuals, while the relative proportions of each progenitor type are maintained. Whether this change in absolute progenitor number has functional consequences for the formation of specific neural circuits in different species is poorly understood. However, it is clear that changes in the relative sizes of progenitor domains in a given species lead to significant developmental defects. Defects in progenitor fate specification lead to defects in the generation of postmitotic neurons that can cause motor or sensory impairment. For instance, p3 progenitor misspecification causes loss of V3 neurons and defects in the rhythmicity and gait of locomotion (Holz et al., 2010; Zhang et al., 2008). To better understand the significance of pattern scaling between species, it is necessary to find and quantitatively analyze examples where scaling is imperfect between species.

## 7 CONCLUDING REMARKS

The question of why elephants are bigger than mice has been asked many times, almost always accompanied by the surprise of how little we know about the answer (Conlon & Raff, 1999; Stocker & Hafen, 2000). Yet, the knowledge that has been obtained in past decades gets us closer. Multiple studies in the spinal cord have led to an advanced understanding of pattern formation, by deciphering the components as well as dynamic properties of morphogen‐driven gene regulatory networks that are responsible for specifying cell identities (Briscoe & Small, 2015; Lai et al., 2016). This has generated extensive knowledge of the dynamics of morphogen signaling, which is also involved in controlling tissue growth and cell cycle progression. Progress has also been made in understanding how the mode of cell division is controlled and coordinated with the acquisition of cell fate. Furthermore, studies in mouse, chick, and zebrafish have progressed in parallel, offering insight into the spinal cord development of different species. Thus, the spinal cord offers an excellent opportunity to delve deeper into the question of size control.

There is still much to be learned about the individual determinants of tissue growth—initial size, cell cycle progression, cell size and shape, and tissue anisotropy (see Box 1). The role of morphogen signaling, mechanics and other mechanisms in regulating these processes is poorly understood. Perhaps the biggest challenge is to identify in what way different levels of regulation are coordinated to produce a spinal cord with defined species‐specific dimensions. Many tools to tackle this challenge are already available—advanced imaging techniques, culture methods for whole embryos, and ex vivo systems, such as explants and ES cell differentiation, that allow precise manipulation of development. Because the question of size control is inherently quantitative, rigorous mathematical and biophysical descriptions are instrumental for informative experimental design and correct interpretation of the data. Further improvement and establishment of new methods that allow quantification of growth parameters, as well as measurements of signaling and mechanical forces, will greatly help to advance the field.

BOX 1 **KEY QUESTIONS**

- The initial pool of progenitors that gives rise to the spinal cord, the changes in cell number driven by cell proliferation and cell loss, the cell sizes and shapes, and the anisotropy of tissue growth can affect the overall size and shape of the spinal cord. What is the contribution of each of these factors to spinal cord size in different species?
- How do morphogens control the rates of cell proliferation, neuronal differentiation and apoptosis in the spinal cord? What are the molecular targets of morphogen signaling within the machineries that regulate these processes? How does the interpretation of morphogen signaling at the molecular level determine the tissue level dynamics of spinal cord growth?
- How is the dynamics of neuronal differentiation controlled? How is neuronal differentiation molecularly linked to the control of cell cycle length and cell cycle progression? To what extent is the differentiation rate determined by the dynamics of cell intrinsic gene regulatory networks versus cell extrinsic factors?
- How do progenitor cell sizes and shapes change during spinal cord development and how is this regulated?
- How is the anisotropy of tissue growth controlled in the spinal cord? What is the role of mechanical forces versus biochemical signaling in this process?
- How is spinal cord size sensed and corrected during development? What are the common and what are the distinct features of growth control systems in different species?
- How is spinal cord pattern coordinated with its size? How do different species achieve pattern scaling between differently sized individuals?

## CONFLICT OF INTEREST

The authors have declared no conflicts of interest for this article.

## AUTHOR CONTRIBUTIONS

**Katarzyna Kuzmicz‐Kowalska:** Conceptualization; investigation; writing‐original draft; writing‐review and editing. **Anna Kicheva:** Conceptualization; funding acquisition; investigation; project administration; supervision; writing‐original draft; writing‐review and editing.

## RELATED WIREs ARTICLES

The wing and the eye: A parsimonious theory for scaling and growth control?

Morphogen interpretation: Concentration, time, competence, and signaling dynamics

Molecular genetic control of cell patterning and fate determination in the developing ventral spinal cord
